# A new genus and species of tettigarctid cicada from the early Miocene of New Zealand: *Paratettigarcta zealandica* (Hemiptera, Auchenorrhyncha, Tettigarctidae)

**DOI:** 10.3897/zookeys.484.8883

**Published:** 2015-03-06

**Authors:** Uwe Kaulfuss, Max Moulds

**Affiliations:** 1 Department of Geology, University of Otago, PO Box 56, Dunedin 9054, New Zealand; 2 Department of Entomology, Australian Museum, 6 College Street, Sydney, NSW 2010, Australia

**Keywords:** Cicadoidea, Tettigarctidae, Miocene, New Zealand, Hindon Maar, Otago

## Abstract

A new genus and species of primitive cicada (Hemiptera: Tettigarctidae) is described from the early Miocene of southern New Zealand. *Paratettigarcta zealandica* **gen.** et **sp. n.** is the first cicada (Cicadoidea) fossil from New Zealand and exhibits wing venation patterns typical for the subfamily Tettigarctinae. It differs from other fossil taxa and the extant genus *Tettigarcta* in the early divergence of CuA2 from the nodal line in the forewing, its parallel-sided subcostal cell, the early bifurcation of vein M and long apical cells of the hindwing, and in wing pigmentation patterns.

## Introduction

Tettigarctidae (hairy cicadas) is the sister-group to singing cicadas (Cicadidae) from which they are distinguished by various morphological characters such as the greatly expanded pronotum (concealing much of the mesonotum), timbals present in both sexes, a completely developed forewing venation with the radial sector arising near the wing base and veins 1A and 2A separated, and a well-developed, conspicuous nodal line on the forewing (Evans 1941, Moulds 2005). Lacking tympanal auditory organs and possessing only rudimentary timbals, Tettigarctidae are not capable of producing the characteristic sound of singing cicadas – their acoustic signals are instead substrate-transmitted (Claridge et al. 1999). While singing cicadas are known since the Paleocene (Cooper 1941) and comprise about 2,000 extant species on all continents (except Antarctica), Tettigarctidae is a mainly Mesozoic radiation that is represented by just two extant species of *Tettigarcta* White, 1845: *Tettigarcta crinita* Distant, 1883 in southeast Australia and *Tettigarcta tomentosa* White, 1845 in Tasmania (Shcherbakov 2009, Moulds 2012).

The fossil record of family Tettigarctidae (summarised in Shcherbakov 2009 and Moulds in prep.) includes 19 extinct genera in subfamilies Cicadoprosbolinae Bekker-Migdisova and Tettigarctinae Distant mainly from terminal Triassic to Upper Cretaceous strata in the Northern Hemisphere. There are three Paleogene records, one from the Paleocene of Menat, France (*Meuniera haupti* Piton, 1940) and the Eocene of Scotland (*Eotettigarcta scotica* Zeuner, 1944) and Germany (Tettigarctidae gen. et sp. indet.; Wappler 2003), the latter representing the youngest fossil record of Tettigarctidae to date. The only Southern Hemisphere fossils of Tettigarctidae are *Architettix compacta* Hamilton, 1990 and *Tettagalma striata* Menon, 2005 from the Lower Cretaceous (Aptian) Santana Formation in Brazil, with *Magrebarcta* [*Liassotettigarcta*] *africana* Nel, Zarbout, Barale & Philippe, 1998 from the Lower Cretaceous (Aptian) in Tunisia complementing the meagre record from Gondwana.

Here we describe *Paratettigarcta zealandica* gen. et sp. n. as the first cicada fossil from New Zealand and, as it is of early Miocene age, the youngest fossil record of Tettigarctidae. This new genus and species comes from a newly discovered paleontological site at Hindon Maar in southern New Zealand and a brief discussion is presented of the depositional setting and the age of the locality as it is currently known.

## Locality and age

**Figure 1.**
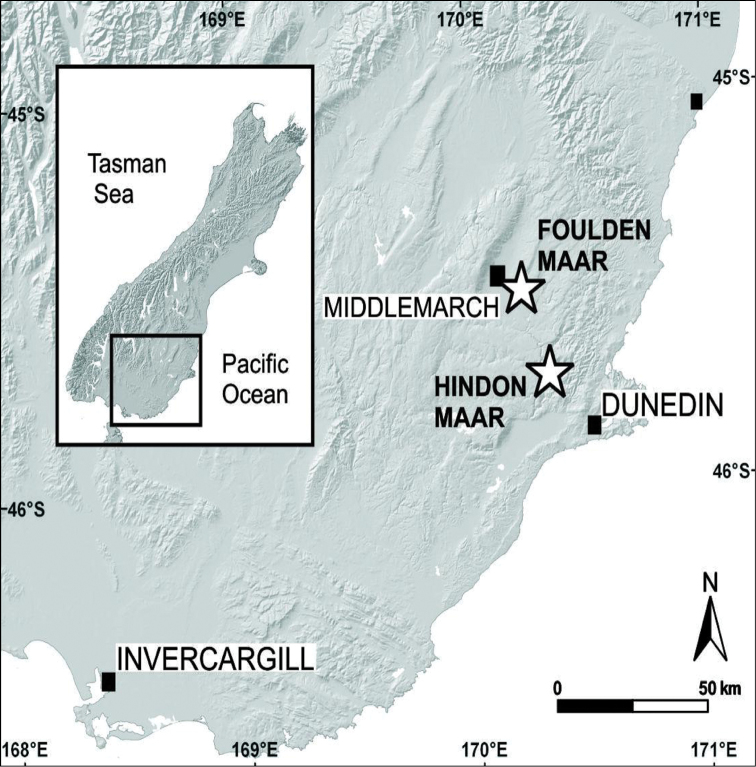
Map showing the position of the new fossil locality at Hindon Maar on the South Island of New Zealand (see text for explanation).

Hindon Maar is a new paleontological site in Otago, South Island, New Zealand, ~10 km N of Outram, near Dunedin (45°45.62'S; 170°15.88'E; Fig. 1). Hindon Maar is located in the southern part of the Waipiata Volcanic Field, which comprises about 150 volcanic remnants of maar-diatremes, scoria cones, plugs, dikes and lava flows (Coombs et al. 2008). The fossil site is situated on private farmland within a shallow, semi-circular topographic depression (500×800 m in diameter) cut into regional metamorphic basement (Otago Schist of Jurassic age). The topographic basin coincides with an aeromagnetic high that is likely to indicate the presence of volcanic material at some depth below surface (data from Glass Earth Gold/Otago Regional Council; released in 2011). Bedded volcanoclastic rocks exposed at the margin of the basin are presumably the remnants of a largely eroded tephra rim; these have yet to be studied and mapped in detail (pers. obs.).

Youngson (1993) reported a 15 m thick sequence of coarse-grained schistose siliciclastics overlain by diatomaceous and carbonaceous laminites from a nearby site, and proposed deposition in a maar lake. Temporary excavations by the Geology Department, University of Otago, at two sites within the basin in early 2014 encountered two lacustrine facies associations beneath 1–2 m of Quaternary loess and alluvium. One facies represents a >3 m thick, thinly (? seasonally) laminated, fossiliferous diatomite interbedded with graded or massive diatomaceous mass-flow beds, which is very similar to that described from the earliest Miocene Foulden Maar fossil Lagerstätte, 26 km NNW of Hindon (Lindqvist and Lee 2009). The second facies is a >2.5 m thick, dark-brown, thickly laminated, carbonaceous mudstone containing cm-thick intervals of thinly laminated intervals. Both facies types are clearly of lacustrine origin, as indicated by siliceous limnic microfossils such as pennate diatoms, siliceous spicules of freshwater sponges (*Spongilla* sp.), and chrysophycean cysts. The thickly laminated mudstone in which the cicada fossil was found contains a well-preserved, diverse terrestrial biota, including spores/pollen, leaves, flowers, fruits, seeds and cones from ferns, conifers and several angiosperm families (JM Bannister pers. comm. 2014, DC Mildenhall pers. comm. 2014), numerous coprolites (presumably from waterbirds), juvenile and adult galaxiid fish, and insects of the orders Hemiptera, Coleoptera, Hymenoptera and Trichoptera (pers. obs.). Preserved leaf and insect cuticles and soft tissue of fish, as well as the laminated biogenic sediment showing no signs of bioturbation all suggest deposition in a deep, presumably anoxic lacustrine environment.

The circular topographic basin, coinciding with an aeromagnetic high and situated in a monogenetic volcanic field, as well as the bedded pyroclastic deposits at the basin margin are all features typically associated with partly eroded maar-diatreme volcanoes, while the fine-grained laminated, biogenic sediment argues for (but is not restricted to) deposition in a maar-lake. The geological and paleontological evidence that is currently available thus suggests that Hindon Maar is a maar-type fossil lagerstätte that may contribute significantly to our understanding of Neogene Australasian biodiversity in the future.

The early Miocene age of Hindon Maar is based on palynology of the lacustrine sediments and on radiometric ages previously published for Waipiata volcanic rocks. K-Ar and ^40^Ar/^39^Ar ages provided by Coombs et al. (2008) indicate that Waipiata volcanism lasted from 24.8±0.6 to 8.9±0.9 Ma, which represents the time range in which the Hindon Maar very likely erupted. The palynological assemblage in the lacustrine diatomite at Hindon indicates an early Miocene age (Aquitanian/Burdigalian; 23.03–15.97 Ma) corresponding to New Zealand stages Otaian to Altonian (DC Mildenhall in Youngson 1993), with a maximum age close to the Duntroonian–Waitakian boundary, as indicated by the presence of *Coprosma* pollen (DC Mildenhall pers. comm. 2014). The fossil biota from Hindon Maar might therefore be coeval with or slightly younger than that of the Foulden Maar fossil lagerstätte, which has been dated at 23 Ma (Lindqvist and Lee 2009).

## Material and methods

The studied fossil comprises overlapping fragments of a hind and forewing preserved as part and counterpart (Fig. 3A, B). The venation in basal parts of both wings, and that of the distal part of the forewing, is not preserved; the wing outline is decipherable for the distal margin of the hindwing only. Forewing and hindwing have mostly separated when the sediment was split open; as a consequence, venation of the hindwing is mainly visible on the part (together with faint traces of forewing venation; Fig. 3A) and venation of the forewing is mainly visible on the counterpart (Fig. 3B). The insect body and its appendages are not preserved.

Photomicrographs were taken with a Canon T3 camera attached to a Nikon SMZ1000 stereomicroscope. Wetting the specimen with ethanol accentuated the visibility of venation patterns and outlines of the wings. Photomicrographs taken at several depths of field were stacked using Photoshop CS5.1 software (Adobe Systems Inc.). Our terminology of wing venation and cells follows that of Moulds (2005) (see Fig. 1). The specimen is stored in a refrigerator (in order to prevent desiccation of the mudstone matrix) in the Geology Department, University of Otago under catalogue number OU45476.

## Systematic paleontology

### Family Tettigarctidae Distant, 1905 Subfamily Tettigarctinae Distant, 1905

#### Paratettigarcta gen. n.

Taxon classificationAnimaliaHemipteraTettigarctidae

http://zoobank.org/9AD59A6E-DDE2-4ED7-AC62-BB88EE02C988

##### Type species.

*Paratettigarcta zealandica* new species, designated herein (Figs 3, 4). No other species are currently included in the genus.

##### Diagnosis.

*Paratettigarcta* is most similar in hindwing venation to that of *Eotettigarcta* Zeuner, 1944 from the Paleocene of the United Kingdom (*Eotettigarcta* is known only from a partial hindwing) but differs in its more parallel-sided subcostal cell (the most anterior of the distal cells) where RA lies parallel to Sc for most of its length rather than gradually diverging, and in the branching of vein M where M_1_ branches before M_3_ (after in *Eotettigarcta*). There are also similarities in the forewing of *Paratettigarcta* with extant *Tettigarcta* from which *Paratettigarcta* differs in the early divergence of CuA_2_ from the nodal line in the forewing (late divergence in *Tettigarcta*). The hindwing of *Paratettigarcta* is quite different from that of *Tettigarcta*, especially in the apical cells that are much longer than those of *Tettigarcta*, in particular the anterior most cell (subcostal cell) that is wide and extended far beyond crossvein r (narrow and only a little extended beyond r in *Tettigarcta*). Further, *Paratettigarcta* has pigmented wing patterns not unlike those present in *Eotettigarcta* (and some other fossil Tettigarctidae) but such patterns are absent in extant *Tettigarcta*.

##### Description.

Forewing veins R and M branched close to base of forewing so that ulnar cells u1-u3 and medial cell are long and narrow; vein CuA strongly bowed before branching. Nodal line clearly defined and departing the extremity of vein CuA_2_. Crossvein r-m nearly straight, steeply angled to RP and M_1_; m gently bowed, almost perpendicular to M_2_ and M_3_; m-cu strongly bowed, meeting M_4_ nearly perpendicularly and meeting CuA_1_ at a steep angle. Hindwing apical cells tending long and narrow, a1 almost as long as a2 so that crossvein r meets RA within its proximal quarter; Sc and RA wide apart, almost as wide as width of apical cell 1.

##### Etymology.

The genus name is a combination of *para* (Latin from Greek, meaning “near”) and the extant genus-group name *Tettigarcta*.

#### Paratettigarcta zealandica sp. n.

Taxon classificationAnimaliaHemipteraTettigarctidae

http://zoobank.org/2EB7CDEB-D467-4F04-9432-378176CB74D6

Figs 3 , 4

##### Diagnosis.

**Figure 2.**
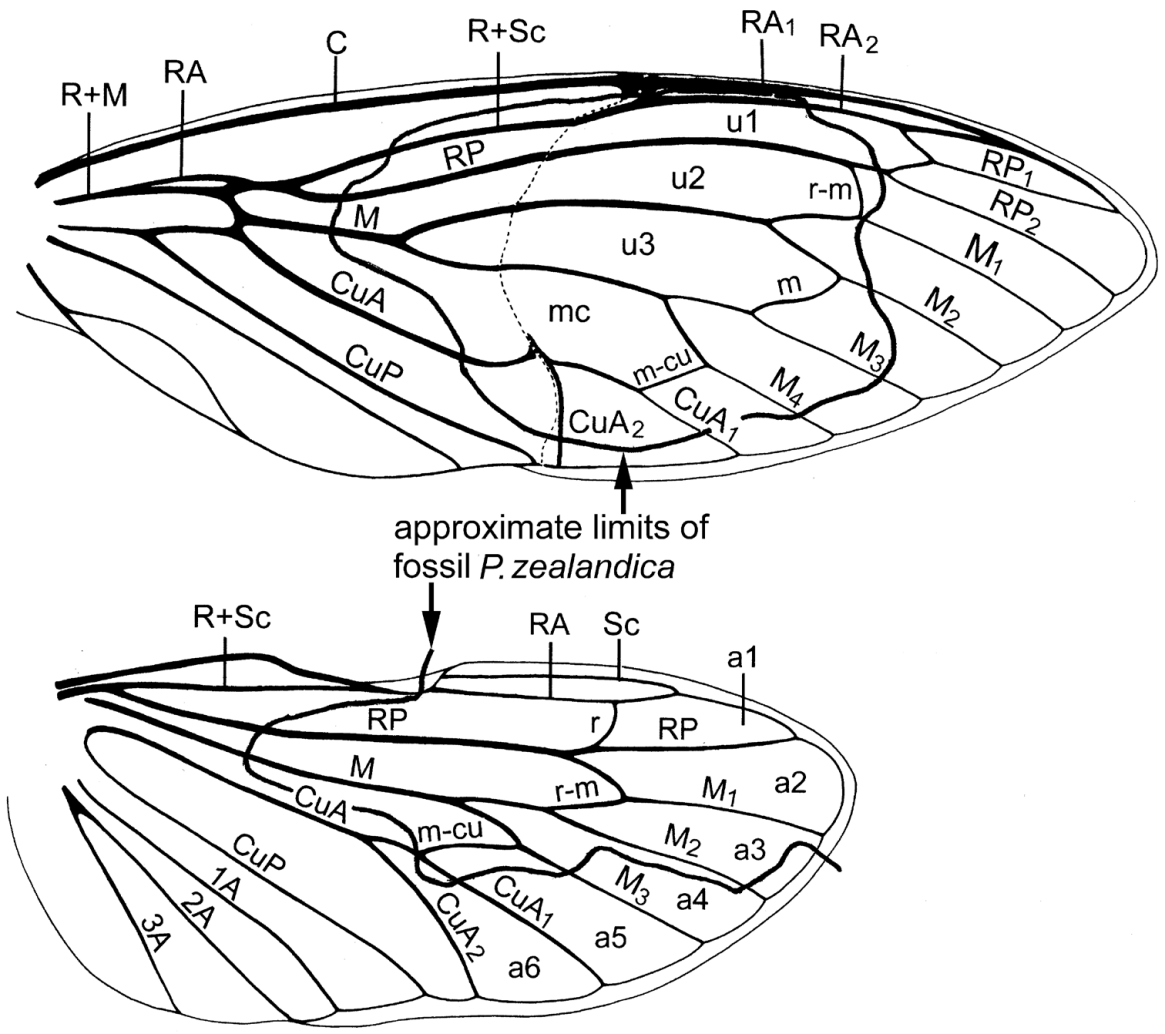
*Tettigarcta crinita* Distant, fore and hindwings; areas of the wings preserved in the new fossil species are indicated (modified from Moulds 2005). **A** anal vein **a** apical cell **C** costal vein **CuA** cubitus anterior vein **CuP** cubitus posterior vein **M** median vein **m** medial crossvein **mc** medial cell **m-cu** mediocubital crossvein **nl** nodal line **R** radius **r** radial crossvein **RA** radius anterior **r-m** radiomedial crossvein **RP** radius posterior **Sc** subcostal vein **u** ulnar cell.

*Paratettigarcta zealandica* sp. n. differs from other Tettigarctidae by the attributes discussed in the generic diagnosis above. In particular the forewing of *Paratettigarcta zealandica*, that is remarkably similar to extant *Tettigarcta crinita* Distant, 1883 (Fig. 2) and *Tettigarcta tomentosa* White, 1845 (the only described species of *Tettigarcta*), differs as follows: (a) forewing crossvein m gently bowed and almost perpendicular to M_2_ and M_3_ rather than steeply angled and broadly ‘S’-shaped; (b) forewing crossvein m-cu strongly bowed rather than nearly straight; (c) hindwing apical cells long and narrow, much longer than those of extant *Tettigarcta* (compare Figs 2 and 4).

**Figure 3.**
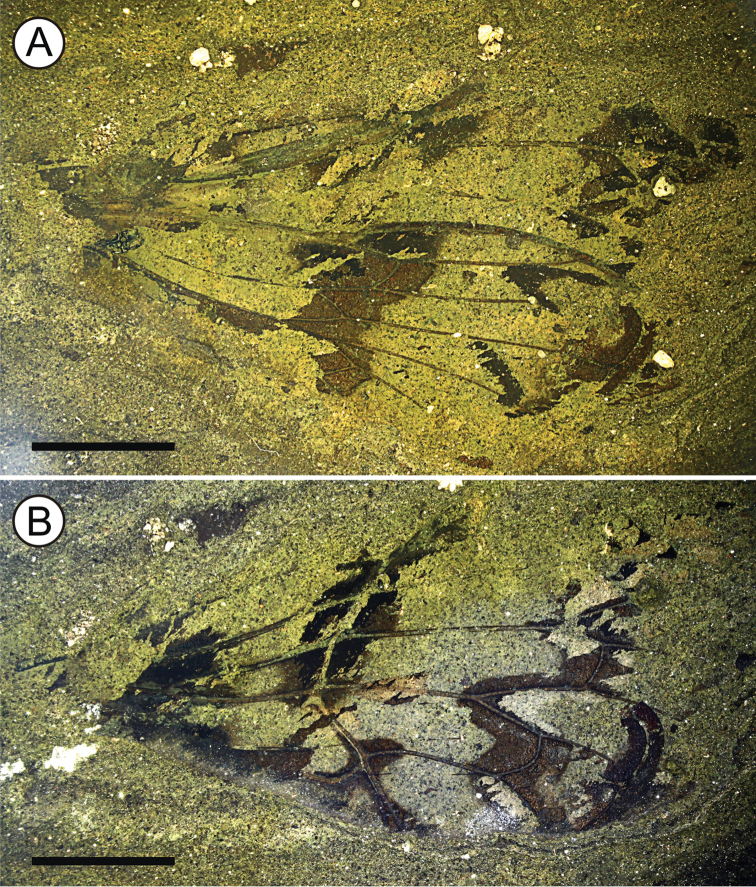
Photomicrographs of *Paratettigarcta zealandica* gen. et sp. n., holotype OU45476, fore and hindwing **A** part and **B** counterpart (mirror inverted), photographed under ethanol. Scale bar: 5 mm.

**Figure 4.**
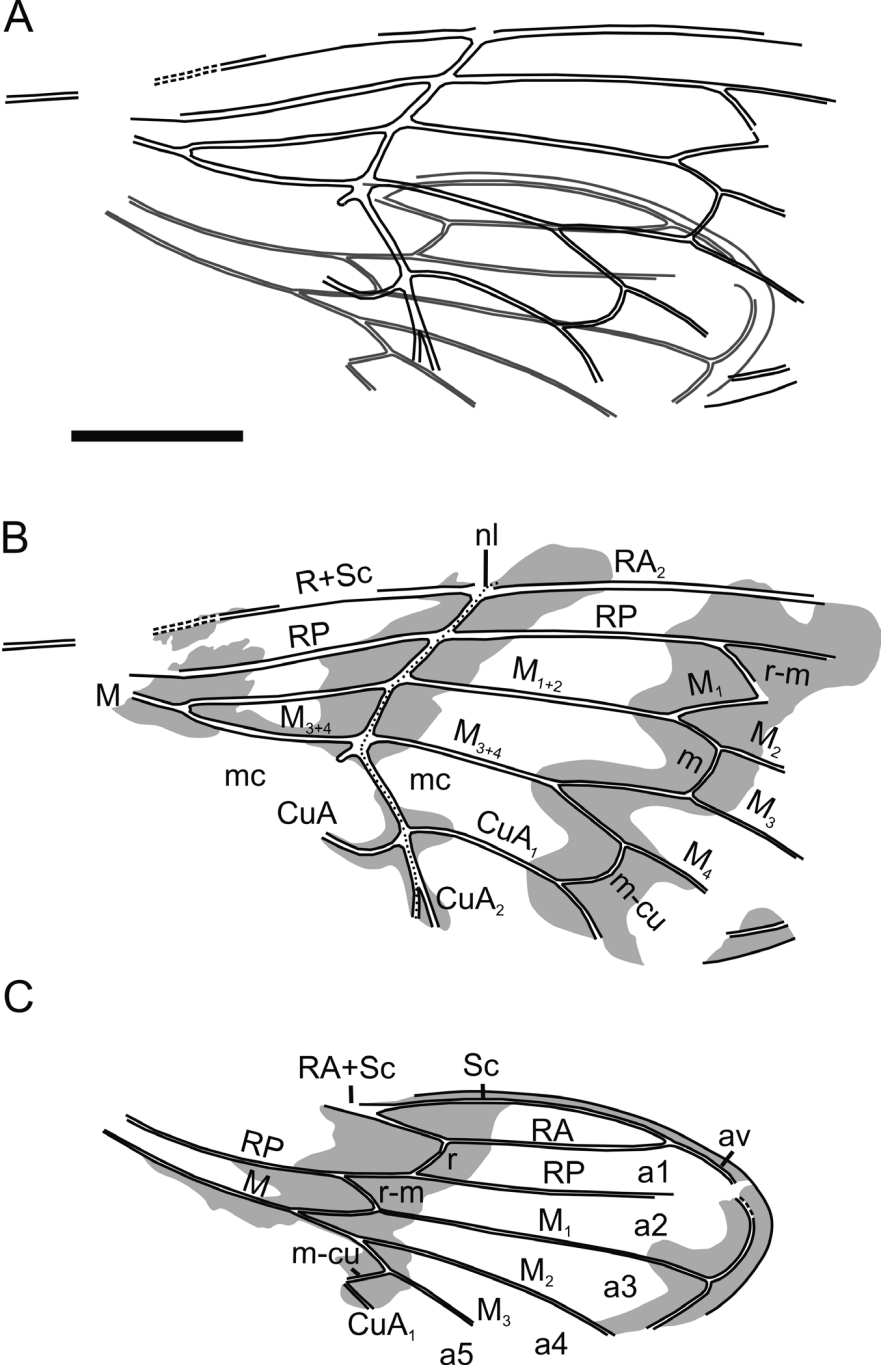
Line drawings of *Paratettigarcta zealandica* gen. et sp. n., holotype OU45476 **A** overlapping fore and hindwing as preserved **B** forewing and **C** hindwing, pigmented areas shown as preserved. **A** apical cell **av** ambient vein **C** costal vein **CuA** cubitus anterior vein **CuP** cubitus posterior vein **M** median vein; medial crossvein **mc** medial cell **m-cu** mediocubital crossvein **nl** nodal line **R** radius **r** radial crossvein **RA** radius anterior **r-m** radiomedial crossvein **RP** radius posterior **Sc** subcostal vein. Scale bar: 5 mm.

##### Description.

Holotype. *Forewing* similar to extant *Tettigarcta* in size, shape and venation (compare Figs 2, 4). Impression 24.3 mm maximum length by 10.2 mm maximum width. Bearing dark pigmented transverse bands, one near the wing base, one following the nodal line between costa and M_3+4_, one following the bases of apical cells, and one along apical margin. Nodal line strongly defined; CuA strongly bowed before forking at nodal line; crossvein r-m nearly straight and angled to both RP and M_1_; crossvein m gently bowed, nearly perpendicular to M_2_ and M_3_; crossvein m-cu strongly bowed, meeting M_4_ nearly perpendicularly, meeting CuA_1_ at a steep angle. *Hindwing* impression 19.5 mm maximum length by 7.3 mm maximum width. Bearing dark bands, one from near coupling lobe to at least crossvein m-cu, and a dark streak behind M stem. Marginal membrane well developed but not exceedingly broad. Crossvein r nearly straight and angled to both RA and RP; crossvein r-m nearly straight and steeply angled to both RP and M; crossvein m-cu straight and angled to both M_3_ and CuA_1_.

##### Type-specimen.

Holotype OU45476, hind and forewing from lacustrine mudstones at Hindon Maar (early Miocene; I44/f0392 in the New Zealand Fossil Record File), Waipiata Volcanic Field, 10 km N of Outram, Otago, southern New Zealand; deposited in the Department of Geology, University of Otago.

##### Etymology.

The species name refers to New Zealand, where this species was distributed in the Miocene.

##### Comments.

*Paratettigarcta zealandica* gen. et sp. n. appears closest to *Eotettigarcta scotica* based on the hindwing venation (the latter known only from a partial hindwing). In particular Sc and RA are widely separated, and it is likely that the apical cells are of similar length with a1 being almost as long as a2. If that is so then *Paratettigarcta* is best placed in the tribe Protabanini of the subfamily Tettigarctinae, family Tettigarctidae, following the classification of Shcherbakov (2009). At around 23–16 Ma this would make *Paratettigarcta zealandica* the youngest known Tettigarctidae fossil, the next youngest at 44 Ma being an undescribed Tettigarctinae tentatively placed in the tribe Tettigarctini (Wappler 2003, Shcherbakov 2009).

The forewing venation of *Paratettigarcta zealandica* shows a clear affinity with that of extant species of *Tettigarcta* of which there are only two closely related species, *Tettigarcta crinita* and *Tettigarcta tomentosa* (Moulds 1990). The overall branching of veins and therefore cell proportions are remarkably similar (compare Figs 2, 4). This confirms the placement of *Paratettigarcta zealandica* in the subfamily Tettigarctinae and similar dark wing patterns are found in some extinct genera of this subfamily (e.g. *Liassocicada* Bode, 1953).

## Discussion

The discovery of a *Paratettigarcta zealandica* at Hindon Maar in southern New Zealand documents the presence of family Tettigarctidae in Australasia in the early Miocene. It thus partially fills the spatial and temporal gap that existed between the next youngest Tettigarctidae fossil from the mid-Eocene of Germany (Wappler 2003), tentatively placed into Tettigarctini (Shcherbakov 2009), and the two surviving members of this relict family in southeastern Australia and Tasmania. Extant cicadas of New Zealand comprise 34 endemic species in five genera (all placed in family Cicadidae, tribe Cicadettini), which occur in a wide range of habitats from lowland coastal areas to alpine zones (Larivière et al. 2010). Molecular phylogenetic studies suggested that the extant fauna is the result of two relatively recent (~12 Ma) transoceanic dispersal events from Australia and New Caledonia and subsequent divergence related to the Southern Alps orogeny and glaciations within the last 5 Ma (Arensburger et al. 2004). *Paratettigarcta zealandica* described herein is the first cicada fossil from New Zealand and, although not in family Cicadidae, indicates the presence of cicadas (Cicadoidea) in New Zealand prior to the more recent incursions and radiations that formed the modern fauna. It establishes the relict family Tettigarctidae in southern New Zealand in the early Miocene and documents the extinction of this hemipteran family in New Zealand since then.

By the early Miocene, New Zealand had been an isolated island landmass for at least 57 My, following separation from Australia in the Late Cretaceous. Two biogeographical scenarios can consequently be hypothesised to explain the occurrence of Tettigarctidae at Hindon Maar: (1) colonization of New Zealand via trans-oceanic dispersal of members of this family in or before the early Miocene, for example from Australia or New Caledonia, as proposed for the Cicadidae (Arensburger et al. 2004), or (2) a vicariance origin in which *Paratettigarcta zealandica* evolved from a Gondwanan lineage that had been present in New Zealand since it separated from neighbouring landmasses. The validity of either hypothesis can only be tested by additional finds of Tettigarctidae fossils in the future.

## Supplementary Material

XML Treatment for Paratettigarcta

XML Treatment for Paratettigarcta zealandica
